# Structural characterization of anti-CRISPR protein AcrIE9

**DOI:** 10.1063/4.0000789

**Published:** 2025-12-24

**Authors:** Jeehee Kang, Jasung Koo, Hyejin Oh, Euiyoung Bae

**Affiliations:** 1 Department of Agricultural Biotechnology, Seoul National University, Seoul 08826, Republic of Korea; 2 Research Institute of Agriculture and Life Sciences, Seoul National University, Seoul 08826, Republic of Korea

## Abstract

The arms race between bacteria and bacteriophages has driven the evolution of both CRISPR-Cas systems and anti-CRISPR (Acr) proteins. AcrIE9, a type I-E Acr protein identified in *Pseudomonas aeruginosa*, inhibits Cascade-mediated DNA binding by interacting with the Cas7e subunit. However, its structural basis and precise inhibitory mechanism have remained unclear. Here, we report the crystal structure of AcrIE9 at 1.73 Å resolution, along with additional structural and biochemical analyses. AcrIE9 exists as both monomer and dimer in solution, while the crystal structure reveals a homodimeric assembly. Each protomer adopts a unique α/β architecture, and structural similarity searches indicate that AcrIE9 represents a previously uncharacterized protein fold. *In vitro* binding assays using individually purified type I-E Cas subunits from *P. aeruginosa* did not detect direct interaction with AcrIE9, including with Cas7e. These findings suggest that AcrIE9 may recognize a composite interface formed only within the intact Cascade complex, consistent with the AlphaFold3 prediction of multivalent interactions with Cas7e subunits. Taken together, this study provides the structural characterization of AcrIE9 and supports an inhibitory mechanism involving a multi-subunit binding surface on Cascade.

## INTRODUCTION

The coevolution between bacteria and bacteriophages (phages) has driven the development of diverse defense and counter-defense systems based on a variety of molecular mechanisms.^1,2^ Among these, the CRISPR-Cas system is a well-established form of prokaryotic adaptive immunity that utilizes acquired foreign nucleic acid fragments as guides to recognize and degrade invading genetic elements from phages and plasmids.^3,4^ CRISPR-Cas systems are classified into two classes, each comprising three types and multiple subtypes. In class 1 systems, interference is mediated by a multi-subunit complex composed of several Cas proteins, whereas class 2 systems rely on a single, multidomain Cas protein.^5,6^

Among class 1 CRISPR-Cas systems, type I is the most widely distributed, and type I-E is one of its well-characterized subtypes.^5–8^ The type I-E system consists of eight distinct Cas proteins. Cas1 and Cas2 are responsible for integrating foreign nucleic acid fragments into the CRISPR array.^7,9^ Five Cas proteins (Cas5e, Cas6e, Cas7e, Cas8e, and Cas11) assemble with a CRISPR RNA (crRNA) to form the CRISPR-associated complex for antiviral defense (Cascade), which functions as the surveillance complex.^7,10–12^ The nuclease Cas3 is then recruited by Cascade to degrade the target DNA.^7,13,14^

Despite the remarkable efficiency of CRISPR-Cas systems, phages have evolved anti-CRISPR (Acr) proteins as a counter-defense strategy.^15–17^ To date, ten distinct Acr proteins (AcrIE1–10) targeting type I-E CRISPR-Cas systems have been identified from phages, prophages, and other mobile genetic elements.^15,18^ Several type I-E Acr proteins (AcrIE1, AcrIE2, AcrIE3, AcrIE4, AcrIE7, and AcrIE10) have been structurally and functionally characterized.^18–23^ These proteins exhibit diverse inhibitory mechanisms by targeting distinct Cas components at different stages of the CRISPR-Cas immune response.^15–17^

AcrIE9 was originally identified in *Pseudomonas aeruginosa*, and its inhibitory function was validated using a CRISPR interference (CRISPRi) transcriptional repression assay in a Δ*cas3* background, suggesting that it blocks DNA binding by the Cascade complex.^24^ More recently, Taranenko *et al.* reported that AcrIE9 specifically interacts with the Cas7e subunit of Cascade, thereby preventing its association with DNA.^25^ However, the molecular structure of AcrIE9 has remained unknown. In this study, we report the crystal structure of AcrIE9 at a resolution of 1.73 Å and provide structural and biochemical insights into its mechanism of action.

## MATERIALS AND METHODS

### Cloning, expression, and purification

A synthetic *acrIE9* gene was cloned into the pET28a vector containing an N-terminal (His)_6_-maltose binding protein (MBP) tag followed by a tobacco etch virus (TEV) protease cleavage site and a flexible linker with a sequence of (Gly-Ser)_3_ (supplementary material Table S1 and Fig. S1).^26^ The plasmid was transformed into *Escherichia coli* BL21(DE3) cells, which were cultured in lysogeny broth at 37 °C until the optical density at 600 nm reached 0.6. Protein expression was induced with 0.5 mM isopropyl β-d-1-thiogalactopyranoside at 17 °C for 16 h. Cells were harvested by centrifugation and resuspended in buffer [500 mM NaCl, 20% (w/v) glycerol, 5 mM β-mercaptoethanol (BME), 30 mM imidazole, 0.02% (w/v) Triton X-100, 0.3 mM phenylmethanesulfonyl fluoride (PMSF), 20 mM 4-(2-hydroxyethyl)-1-piperazineethanesulfonic acid (HEPES) pH 7.0]. After sonication and centrifugation, the supernatant was loaded onto a 5 ml HisTrap HP column (Cytiva, USA) pre-equilibrated with buffer [500 mM NaCl, 20% (w/v) glycerol, 5 mM BME, 30 mM imidazole, 20 mM HEPES pH 7.0]. Following a wash step, bound proteins were eluted using a linear imidazole gradient up to 500 mM. The (His)_6_-MBP tag was cleaved by TEV protease in buffer [500 mM NaCl, 20% (w/v) glycerol, 5 mM BME, 20 mM HEPES pH 7.0] at 4 °C for 16 h, and separated with the HisTrap HP column. The TEV protease was expressed from the Addgene plasmid (#8827) and purified as described previously with minor modifications.^27,28^ Because TEV protease carries its own (His)_6_ tag, it was retained on the HisTrap HP column. Further purification was performed by size-exclusion chromatography (SEC) using a HiLoad 26/600 Superdex 75 pg column (Cytiva) equilibrated with buffer [300 mM NaCl, 5% (w/v) glycerol, 2 mM 1,4-dithiothreitol (DTT), 20 mM HEPES pH 7.0]. The purified protein was concentrated, flash-frozen in liquid nitrogen, and stored at −80 °C until use.

Type I-E *cas* genes were amplified by polymerase chain reaction from the genomic DNA of *P. aeruginosa* PRD-10 and cloned into the pET28a vector containing an N-terminal (His)_6_-MBP tag (supplementary material Table S1 and Fig. S1). The constructs were transformed into *E. coli* BL21(DE3) cells. Protein expression was induced as described above for AcrIE9. Cells were harvested by centrifugation and resuspended in buffer [500 mM NaCl, 20% (w/v) glycerol, 5 mM BME, 30 mM imidazole, 0.02% (w/v) Triton X-100, 0.3 mM PMSF, 20 mM HEPES pH 7.0]. After sonication and centrifugation, the supernatants were loaded onto a 5-ml HisTrap HP column (Cytiva) pre-equilibrated with buffer [500 mM NaCl, 20% (w/v) glycerol, 5 mM BME, 30 mM imidazole, 20 mM HEPES pH 7.0]. The column was washed with the same buffer, and bound proteins were eluted using a linear gradient of imidazole (up to 500 mM). Final purification was carried out by SEC using a HiLoad 16/600 Superdex 200 pg column (Cytiva), equilibrated with buffer [500 mM NaCl, 10% (w/v) glycerol, 2 mM DTT, 20 mM HEPES pH 7.0].

### Analytical SEC

Analytical SEC was performed using a Superdex 200 increase 10/300 GL column (Cytiva) pre-equilibrated with buffer [150 mM NaCl, 2 mM DTT, 5% (w/v) glycerol, 20 mM HEPES pH 7.0]. Proteins (20 *μ*M each) were incubated at 4 °C for 1 h prior to injection. The column was run at a flow rate 0.5 ml/min. The eluted fractions were analyzed by 15% (w/v) sodium dodecyl sulfate–polyacrylamide gel electrophoresis (SDS-PAGE). Proteins were visualized by Coomassie Brilliant Blue staining.

### X-ray crystallography

Crystals of AcrIE9 were obtained at 20 °C by the hanging-drop vapor diffusion method using a 1.7 mM protein solution in buffer (300 mM NaCl, 2 mM DTT, 20 mM HEPES pH 7.0), mixed in a 1:1 ratio with reservoir solution [200 mM NaCl, 25% (w/v) polyethylene glycol 3350, 100 mM HEPES pH 7.0]. For phase determination by single-wavelength anomalous diffraction, selenomethionine (SeMet)-substituted AcrIE9 was expressed in *E. coli* BL21(DE3) cells grown in M9 minimal medium supplemented with SeMet, as described previously.^29^ The selenomethionyl protein was purified and crystallized under the same conditions as the native protein. Crystals appeared within a day and were harvested and flash-frozen in liquid nitrogen on day six for data collection. X-ray diffraction data were collected at 100 K on beamline 7A of the Pohang Accelerator Laboratory. Diffraction images were processed using HKL2000.^30^ Determinations of selenium positions, density modification, and initial modeling building for the selenomethionyl protein were performed using PHENIX.^31^ The initial model of the selenomethionyl structure was used for phasing of the native structure in PHENIX.^31^ The final structure was completed using alternate cycles of manual fitting in Coot and refinement in PHENIX.^31,32^ The stereochemical quality of the final model was assessed using MolProbity.^33^

### AlphaFold3 modeling

The structure predictions were performed by using the AlphaFold3 server, Version 1.4.5.^34^ All settings were left at their default values. The final output includes five predicted structures, from which we selected the model_0.

## RESULTS AND DISCUSSION

### AcrIE9 forms a homodimer in solution and crystal

**FIG. 1.**
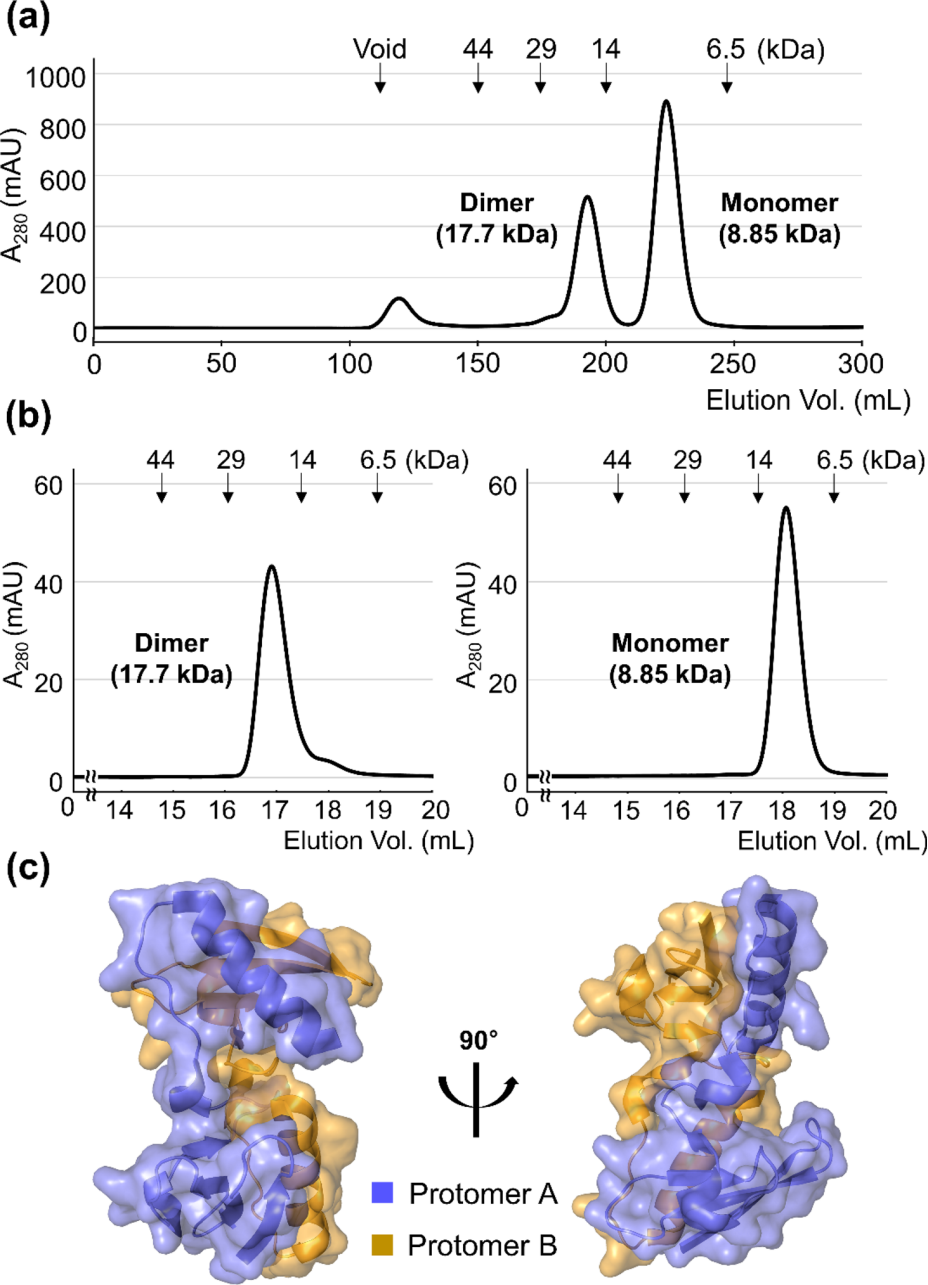
Dimeric assembly of AcrIE9 in solution and in the crystal lattice. (a) Preparative SEC profile of AcrIE9 during purification. Elution volumes of protein standards are indicated, and the calculated molecular weights of the AcrIE9 dimer and monomer are also shown. (b) Analytical SEC profiles of the purified AcrIE9 monomeric and dimeric species of AcrIE9. (c) Dimeric assembly formed by AcrIE9 in the crystal lattice.

During purification, AcrIE9 was eluted as two distinct peaks on a preparative SEC column, corresponding to its dimeric and monomeric states, respectively [Fig. 1(a)]. These two species were separately pooled for further characterization. Subsequent analytical SEC revealed that each species retained its original assembly state, either dimeric or monomeric [Fig. 1(b)], indicating that the dimerization is stable rather than transient. Crystals were obtained from the monomeric fraction of AcrIE9, and its structure was determined at a resolution of 1.73 Å (Table I). Unexpectedly, the asymmetric unit contained two AcrIE9 protomers forming a dimeric assembly within the crystal lattice [Fig. 1(c)], suggesting that dimerization may be promoted under the high protein concentration used for crystallization. Consistently, the Protein Interfaces, Surfaces, and Assemblies (PISA) analysis predicted the dimeric assembly as the probable quaternary structure of AcrIE9.^35^ A total of 29 hydrogen bonds were identified between the two protomers. Upon dimerization, 1919.8 and 1889.9 Å^2^ of solvent-accessible surface area were buried on each protomer, corresponding to 32.5% and 34.0% of their respective total surface areas. Taken together, these findings indicate that AcrIE9 is likely to form a stable homodimer.

**TABLE I.** Data collection, phasing, and refinement statistics.

	Native^a^	Selenomethionyl^a^
Space group	P1	P1
Unit cell parameters (Å)	a = 25.34, b = 37.17, c = 40.70, α = 114.10°, β = 90.10°, γ = 104.70°	a = 25.53, b = 37.67, c = 41.14, α = 65.43°, β = 89.98°, γ = 75.55°
Wavelength (Å)	0.9793	0.9791
**Data collection statistics**		
Resolution range (Å)	30.00–1.73 (1.79–1.73)	50.00–2.20 (2.28–2.20)
Number of reflections	13 091 (1285)	12 517 (1224)
Completeness (%)	95.8 (94.8)	98.0 (96.9)
R_merge_^b^	0.082 (0.719)	0.160 (0.271)
R_meas_^c^	0.097 (0.797)	0.172 (0.310)
Redundancy	3.5 (3.7)	3.2 (2.9)
CC1/2	0.995 (0.816)	0.940 (0.940)
CC*	0.999 (0.948)	0.984 (0.984)
Mean I/σ	8.67 (1.88)	23.98 (11.75)
**Phasing statistics**		
f′, f″ used in phasing		−5.3, 5.8
Figure of merit		0.41
**Refinement statistics**		
Resolution range (Å)	21.53–1.73	
R_cryst_^d^/R_free_^e^ (%)	21.3/24.3	
RMSD bonds (Å)	0.007	
RMSD angles (°)	0.993	
Average B-factor (Å^2^)	27.3	
Protein	26.5	
Water	33.6	
Number of water molecules	120	
Ramachandran favored (%)	97.0	
Ramachandran allowed (%)	3.0	

^a^ Values in parentheses are for the highest-resolution shell.

^b^ R_merge_ = Σ_h_Σ|I_i_(h) − <I(h)>|/Σ_h_Σ_i_I_i_(h), where I_i_(h) is the intensity of an individual measurement of the reflection and <I(h)> is the mean intensity of the reflection.

^c^ R_meas_ = Σ_h_| $\sqrt{n_{h}/n_{h}-1}$Σ|I_i_(h) − <I(h)>| |/Σ_h_Σ_i_I_i_(h), where I_i_(h) is the intensity of an individual measurement of the reflection and <I(h)> is the mean intensity of the reflection.

^d^ R_cryst_ = Σ_h_ǁF_obs_|−|F_calc_ǁ/Σ_h_|F_obs_|, where F_obs_ and F_calc_ are the observed and calculated structure factor amplitudes, respectively.

^e^ R_free_ was calculated as R_cryst_ using ∼5% of the randomly selected unique reflections that were omitted from structure refinement.

The majority of structurally characterized type I-E Acr proteins, including AcrIE2, AcrIE3, AcrIE4, and AcrIE7, function as monomers, whereas AcrIE1 and AcrIE10, like AcrIE9, form dimers (supplementary material Fig. S2).^19–23^ However, the AcrIE1 and AcrIE10 dimers use distinct mechanisms of action. AcrIE1 binds to the Cas3 nuclease and inhibits its cleavage activity, while AcrIE10 interacts with the Cas7e subunits of Cascade, thereby preventing assembly of the surveillance complex.^18,19^ Therefore, it is premature to infer the inhibitory mechanism of AcrIE9 solely based on its dimeric state.

### AcrIE9 adopts a unique α/β fold

**FIG. 2.**
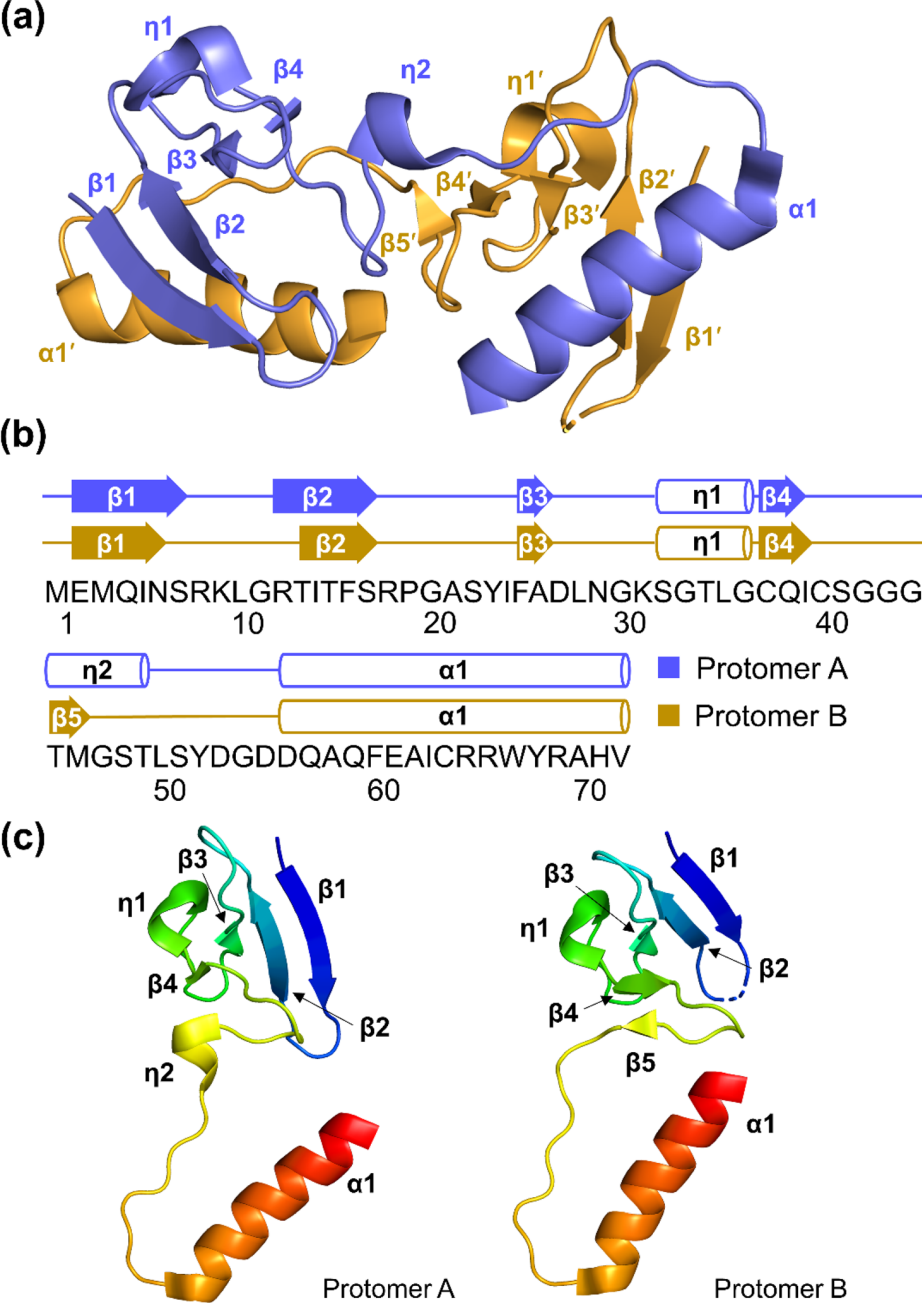
Crystal structure of AcrIE9. (a) Dimeric structure of AcrIE9. The two protomers form a single dimer with pseudo-two-fold symmetry. Secondary structural elements are labeled, and prime symbols denote protomer B. (b) Secondary-structure diagram of AcrIE9. The amino acid sequence is shown and numbered below. (c) Structure comparison of protomer A (left) and protomer B (right). The two protomers display secondary-structure switching in the η2/β5 segment and overall conformational differences. Each protomer is shown in a rainbow gradient from the N terminus (blue) to the C terminus (red).

In the crystal structure, AcrIE9 protomers adopts an α/β architecture consisting of an N-terminal antiparallel β-sheet and a long C-terminal α-helix (Fig. 2). In protomer A, four β-strands form the antiparallel β-sheet in the order β1–β2–β3–β4. Two 3_10_-helices, η1 and η2, are positioned adjacent to the β-sheet. η1 lies along one face of the sheet, whereas η2 is located near β4 on the outer edge. A loop following the η2 helix extends away from the β-sheet, and the long α1 helix runs in the opposite direction, folding back toward the sheet. In protomer B, however, η2 is replaced by an additional β-strand, β5, which interacts with β4 to form a five-stranded β-sheet. This secondary-structure switching appear to account for the conformational difference between the two protomers (Fig. 2 and supplementary material Fig. S3). Structural alignment of the two protomers yielded a root-mean-square deviation (RMSD) of 2.76 Å. In contrast, pairwise RMSD values for the N-terminal (residues 1–44) and C-terminal (residues 50–72) regions, separated by the η2/β5 segment (residues 45–49), were only 0.20 and 0.11 Å, respectively (supplementary material Fig. S4).

A structural similarity search using the DALI server did not identify any significant homologs for either the AcrIE9 protomer or the dimer.^36^ Only partial structural matches were found, with low Z scores and high RMSD values. Interestingly, AlphaFold3 predicted a monomeric structure for AcrIE9 with a predicted template modeling (pTM) score of 0.85 and high predicted local distance difference test (pLDDT) score, indicating high model confidence [supplementary material Figs. S5(a) and S5(b)].^34^ The pTM score reflects the confidence in the global fold of the predicted model, and pLDDT provides residue-level local confidence.^34,37–41^ Structural alignment revealed a notable similarity between the predicted monomeric model and one half of the dimer observed in the crystal structure [supplementary material Figs. S5(c) and S5(d)]. The AlphaFold3 model resembled the N-terminal region of one protomer and, at the same time, the C-terminal region of the other protomer in the dimer, suggesting a possible domain-swapped arrangement.^42,43^ Notably, we previously made a similar observation for another Acr protein, AcrIF2, which behaves as a monomer in solution but crystallizes as a domain-swapped dimer.^44^

On the other hand, the AlphaFold3 prediction of the AcrIE9 dimer yielded a dimeric assembly distinct from the one in our crystal structure [supplementary material Fig. S6(a)].^34^ The pTM and interface predicted template modeling (ipTM) scores were 0.49 and 0.13, respectively, reflecting relatively low confidence in the dimeric model [supplementary material Fig. S6(b)]. The ipTM score estimates the reliability of the predicted interface between protomers within a complex, and values below 0.6 are generally interpreted as indicating very low confidence in the predicted interface.^34,39^ Although the predicted alignment error (PAE) values within each protomer were mostly below 5 Å, the PAE values for residue pairs across the two protomers exceeded 15 Å for the majority of positions [supplementary material Fig. S6(b)], indicating very low confidence in the predicted relative orientation. Because PAE reflects the expected positional error between residue pairs, high inter-protomer PAE values suggest that the model does not reliably capture the spatial relationship between the protomers.^34,37,45^ Taken together, the low ipTM score and the high inter-protomer PAE values indicate that AlphaFold3 does not yield a reliable or plausible dimeric model for AcrIE9. AlphaFold3 also generated several additional dimeric models, all with ipTM scores below 0.20. Notably, the predicted dimerization interfaces differed among these models, further indicating that AlphaFold3 does not identify a consistent or plausible dimeric arrangement. Nonetheless, our SEC results and crystallographic data support the existence of a dimeric state. It is also noteworthy that AcrIE10, which binds to the Cas7e subunit of Cascade like AcrIE9, forms a butterfly-shaped dimer.^18^ Given these contrasting observations, the biologically relevant oligomeric state of AcrIE9 remains uncertain.

### *In vitro* binding assays of AcrIE9 with type I-E Cas proteins

It has previously been demonstrated that AcrIE9 inhibits the DNA-binding activity of the *P. aeruginosa* Cascade complex, which contains five Cas proteins (Cas5e, Cas6e, Cas7e, Cas8e, and Cas11).^24^ Taranenko *et al.* further demonstrated that AcrIE9 interacts with *E. coli* Cas7e.^25^ However, its binding to individual Cas subunits of *P. aeruginosa* Cascade has never been reported. To identify a target Cas component of AcrIE9, we performed analytical SEC experiments using individual Cascade subunits from *P. aeruginosa* and *E. coli* Cas7e. Due to their poor solubility, we used an N-terminal (His)_6_-MBP tag to enhance the solubility and stability of each recombinant Cas protein.^22^ This strategy has previously enabled the successful identification of target subunits for other type I-E Acr proteins, including AcrIE3, AcrIE4, and AcrIE4-F7.^20,21,23^

**FIG. 3.**
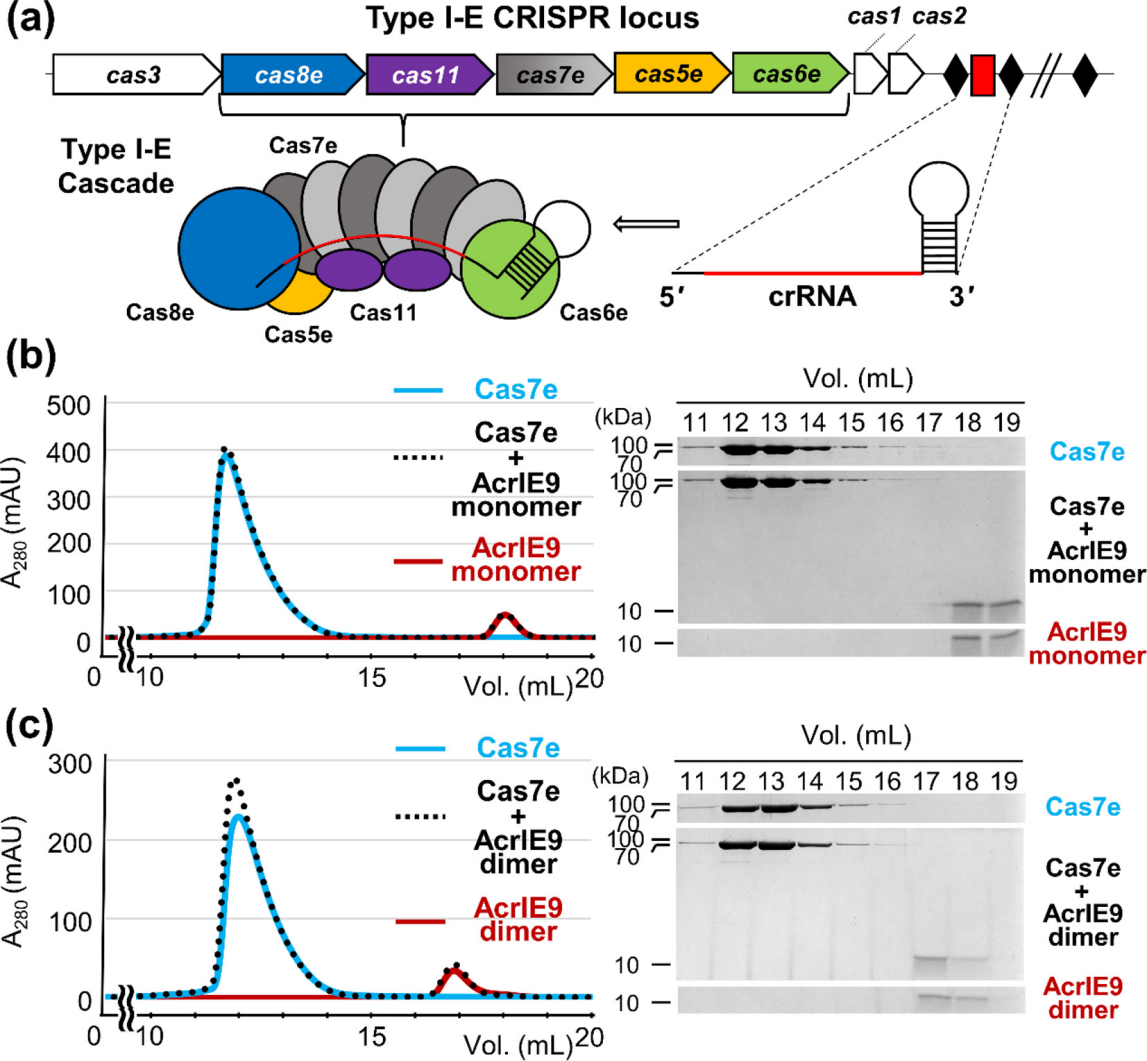
Analytical SEC to assess the interaction between AcrIE9 and Cas7e from *P. aeruginosa.* (a) Schematic representation of the type I-E CRISPR locus and the Cascade complex, a crRNA-guided surveillance assembly. Black diamonds and a red rectangle represent invariable repeats and a variable phage-derived spacer, respectively. (b) and (c) No detectable interaction was observed between (His)_6_-MBP-tagged Cas7e of *P. aeruginosa* and either the monomeric (b) or dimeric (c) form of AcrIE9. Elution fractions were analyzed by SDS-PAGE. Uncropped gel images are provided in Fig. S11.

We first tested the interaction between AcrIE9 and (His)_6_-MBP-tagged *P. aeruginosa* Cas7e. Both dimeric and monomeric forms of AcrIE9 were analyzed, but no shift in elution volume was observed upon mixing with Cas7e, suggesting no detectable interaction (Fig. 3). The chromatograms of the mixtures closely matched the combined profiles of individual AcrIE9 and Cas7e samples. We also obtained essentially identical results from analytical SEC using *E. coli* Cas7e, indicating no detectable interaction between AcrIE9 and the *E. coli* homolog under our experimental conditions (supplementary material Fig. S7). We then extended our binding assays to the other *P. aeruginosa* Cascade subunits (Cas5e, Cas6e, Cas8e, and Cas11), again using both forms of AcrIE9. No interaction was observed with any of the subunits (supplementary material Figs. S8 and S9).

These results were unexpected, given that AcrIE9 has previously been shown to inhibit *P. aeruginosa* type I-E Cascade and to interact with *E. coli* Cas7e.^24,25^ However, we recognized potential limitations in our experimental setup. The (His)_6_-MBP tag (∼42 kDa) may have introduced steric hindrance, potentially interfering with Acr–Cas interactions. Furthermore, the individually expressed recombinant Cas proteins may not adopt their native conformations outside the assembled Cascade complex as proper folding could require co-expression or interactions with neighboring subunits. Alternatively, AcrIE9 may recognize a binding interface formed by multiple Cas7e subunits within the intact Cascade complex, which contains six copies of Cas7e. Indeed, several type I-F Acr proteins, including AcrIF1, AcrIF8, and AcrIF9, have been shown to interact simultaneously with multiple Cas7 homologs in the type I-F Cascade complexes.^15,46,47^ Consistently, the AlphaFold3 model of the AcrIE9-bound *P. aeruginosa* Cascade complex predicted interactions with three distinct Cas7e subunits within 4 Å (supplementary material Fig. S10).^34^ Thus, it is too early to draw definitive conclusions from our binding assays alone regarding the specific target subunit(s) of AcrIE9 within the *P. aeruginosa* type I-E Cascade, underscoring the need for further structural and/or biochemical analyses.

## SUPPLEMENTARY MATERIAL

See the supplementary material for additional details on the structural and biochemical analyses of AcrIE9 (Table S1 and Figs. S1–S11).

## Data Availability

The data that support the findings of this study are openly available in Protein Data Bank, Ref. 48.
